# Diverging effects of mentalization based treatment for patients with borderline personality disorder and schizophrenia: an explorative comparison

**DOI:** 10.3389/fpsyt.2023.1226507

**Published:** 2023-08-25

**Authors:** Jonas G. Weijers, Fleur van Kaam, Jean-Paul Selten, Remco F. P. de Winter, Coriene ten Kate

**Affiliations:** ^1^GGZ Rivierduinen, Institute for Mental Health Care, Leiden, Netherlands; ^2^MHeNs School for Mental Health and Neuroscience, Maastricht University, Maastricht, Netherlands; ^3^Vrije Universiteit Amsterdam, Amsterdam, Netherlands

**Keywords:** mentalization based treatment, mentalizing capacity, borderline personality disorder, schizophrenia, impact of treatment

## Abstract

**Introduction:**

There is robust evidence that both patients with schizophrenia (SCZ) and borderline personality disorder (BPD) display mentalizing difficulties. Less is known however about differences in the way mentalization based treatment (MBT) impacts mentalizing capacity in SCZ and BPD patients. This study compares the impact of MBT on mentalizing capacity in individuals with SCZ and BPD.

**Method:**

The thematic apperception test was used to measure mentalizing capacity. It was administered at the beginning and end of treatment to 26 patients with SCZ and 28 patients with BPD who enrolled in an 18-month long MBT program. For comparison a sample of 28 SCZ patients who did not receive MBT was also included. Using the social cognition and object-relations system, these narratives were analyzed and scored. Missing data was imputed and analyzed using intention-to-treat ANCOVAs with post-treatment measures of mentalizing capacity as dependent variables, group type as independent variable and baseline mentalizing capacities as covariates.

**Results:**

Results showed that patients with BPD showed significantly more improvement on several measures of mentalizing, including complexity of representation (η_p_^2^ = 0.50, *p*_pooled_ < 0.001), understanding of social causality (η_p_^2^ = 0.41, *p*_pooled_ < 0.001) and emotional investment in relationships (η_p_^2^ = 0.41, *p*_pooled_ < 0.001) compared to patients with SCZ who received MBT. No differences were found regarding affect-tone of relationships (η_p_^2^ = 0.04, *p*_pooled_ = 0.36). SCZ patients who received MBT showed greater performance on understanding of social causality (η_p_^2^ = 0.12, *p*_pooled_ = 0.01) compared to SCZ patients who did not receive MBT, but no differences were observed on complexity of representations, capacity for emotional investment or affect-tone of relationships.

**Discussion:**

Patients with BPD performed better after receiving MBT on three dimensions of mentalizing capacity than SCZ patients who received MBT. Remarkably, SCZ patients who received MBT performed better on one dimension of mentalizing capacity compared to SCZ patients who did not receive MBT. Whereas MBT for BPD clearly involves improvement on most aspects of mentalizing, MBT for SCZ seems to thwart a further decline of other-oriented, cognitive mentalizing. Treatment goals should be adapted toward these disorder-specific characteristics.

## 1. Introduction

Schizophrenia-spectrum disorders (SSDs) and borderline personality disorder (BPD) are usually treated as very distinct disorders, both in their respective treatment approaches and the conceptualization of their respective pathogeneses. SSD—an umbrella term comprising different classifications such as brief psychotic disorder, schizoaffective disorder, schizophrenia, and psychotic disorder not otherwise specified—affect around 1.5% of adults and are characterized by episodes of psychosis, which may involve hallucinations or delusions (1). On the other hand, BPD is characterized by instability in interpersonal relationships, self-image, and affect, along with impulsive and reckless behavior, and it affects around 1.6% of adults (2). Whereas BPD is commonly viewed as a pathological development of personality characteristics that hampers functioning and is caused by both biological factors (i.e., temperament) and (childhood) adverse events (3), SSDs are predominantly thought of as a neurodevelopmental disorders [e.g., (4)]. Furthermore, the first choice in treatment for BPD is psychotherapy (3), with pharmacotherapy as an adjunctive component. Some have even argued that treatment for BPD should preferentially be conducted without pharmacotherapy (5). The first choice of treatment in SSDs is still antipsychotic medication, at least regarding positive symptoms like delusions and hallucinations (6).

However, recent research has shown that the distinction between BPD and SSDs is less clear-cut than often assumed and that psychotic disorders exist on a continuum (7). Early views assumed borderline psychopathology occupied a conceptual area between neurosis and psychosis [e.g., (8)], and overlap was by definition expected. Both patients with borderline and psychotic pathology were thought to experience difficulty to differentiate between self- and other generated experiences, with patients with schizophrenia-spectrum disorders also experiencing difficulty distinguishing between fantasy and reality (8). In a recent study, Slotema et al. (9), observed that 38% of patients with a borderline condition also adhered to enough symptoms of an SSD to be given the diagnosis. Thus, there is a greater overlap in symptomatology than previously thought. Both BPD and SSDs are characterized by episodes of disturbed perception of reality, such as hallucinations or delusions. As opposed to SSDs, in BPD such disturbances were, by definition, considered to be transient. However, research has shown that the regularly occurring psychotic symptoms in BPD, including hallucinatory experiences and delusions, also often persist over time, and are for a large part already present in early childhood (10). On the other hand, it is rare for SSD patients to experience hallucinations or delusions continuously, there are often phases of increased intensity and periods of absence. Furthermore, it was previously held that psychotic symptoms in BPD are more related to stress and childhood trauma as opposed to a constitutional vulnerability in SSD. But recent research shows that childhood trauma is a significant causal factor in the development of both disorders (11, 12) and contrary to what was initially thought, both childhood trauma, momentary stress and affective instability play major roles in the severity of psychotic symptoms in patients with SSD (13). Other symptoms that are often observed in both BPD and SSD include mood instability, impulsivity (including substance abuse), and suicidality. Additionally, both patients with BPD and SSDs are thought to experience disturbances in self-awareness and self-representation (14): at times they find it difficult to distinguish between self- or other-generated experiences. Furthermore, whereas SSDs were historically generally treated psychopharmaceutically, several forms of psychotherapy were in fact found to be effective in treating SSDs, including cognitive behavioral therapy for psychosis (15), and eye movement desensitization and reprocessing (16). Moreover, a recent investigation revealed that in young individuals showing the first signs of borderline personality disorder (BPD), there is a notable presence of symptom combinations that closely resemble the early manifestations of bipolar disorders and SSDs and that it is difficult accurately distinguishing these disorders during this early stage and establishing identification frameworks and preventive interventions that are tailored to each specific disorder (17).

A robust body of evidence from the last two decades has also established that both disorders are characterized by disturbances in mentalizing capacity [e.g., (18, 19)]. Mentalizing, or the ability to understand and make sense of one’s own and others’ mental states and emotions, is an important aspect of social cognition. It is the process by which people make sense of each other and themselves, in terms of subjective states and mental processes (20). A recent meta-analysis concluded that BPD patients show impairments in the ability to reflect on their own mind and the mind of others (21). Similarly, several meta-analyses have now established that SCZ patients have an impaired ability to understand thoughts and feelings of others [for overviews see (22, 23)], have an impaired awareness of their own internal sensory-affective experience (24), and show difficulty verbalizing such experience (25). Lastly, separate meta-analyses have concluded that mentalizing capacity is robustly related to psychopathology across psychiatric disorders (26).

Given the widely observed impairments in mentalizing in both disorders and their relation to impaired social functioning and psychopathology, there has been increased interest in treatments that target mentalizing capacity, most notably Mentalization Based Treatment (MBT), the topic of this study (20, 27–30). MBT is a psychodynamic therapy that assists patients in buttressing their reflective capacities. Since its inception (20), MBT has developed into an established treatment for BPD (31). Studies showed that MBT reduces symptomatic burden directly post-treatment, but even years after treatment termination, patients who received MBT continued to show improvement (32, 33). Since these early studies, MBT has been widely implemented as one of the few evidence-based treatments for BPD. Although evidence is still scarce, recent studies suggest that MBT has also beneficial effects on the mentalizing capacity of patients with BPD (28) (Rizzi et al., Under review)^1^ and SSDs (30).

However, whether MBT can be implemented as effectively for the much more narrowly defined classification schizophrenia (SCZ) remains unclear. SCZ is a severe condition that next to positive symptoms, is characterized by negative symptoms, including flattened affect and avolition, disorganized thinking and behavior. There are a few reasons that MBT may not be as impactful for SCZ as for BPD. Firstly, SCZ is generally viewed to be the most severe and chronic disorder among SSDs, and our earlier findings suggest that MBT works less effectively regarding the more chronic variants of SSDs (30). Secondly, despite the symptomatic similarities between BPD and the broad spectrum of SSDs, there is relatively little comorbidity with the much more narrowly defined classification of SCZ—around 2% according to a recent study (9), which points to substantive differences between them. Thirdly, mentalizing difficulties have long been suggested to be more severe in SCZ (34), which has been corroborated by recent research (35–37). Thirdly, it was suggested that mentalizing in BPD seems to be characterized more by an instability rather than a deficit, while patients with SCZ tend to show a more structural impairment (30). Fourthly, BPD patients seem to be characterized by a tendency to excessively attribute incorrect intentions to others, or to “hypermentalize,” and some patients were even observed to perform better at certain tasks of affect-oriented mentalizing compared to healthy controls (38). SCZ patients, in contrast, have been thought to hypomentalize [i.e., to reason unimaginatively and concretely about other person’s behavior; (39)], with their performance on mentalizing tasks being similar to those of autistic patients (18). It should be noted however, that this tendency to hypomentalize seems most prominent in SCZ patients characterized by negative and disorganized symptoms as opposed to those characterized by positive symptoms, who do tend to hypermentalize (40, 41). Lastly, although the evidence is still limited, recent studies have shown that neurocognition (42, 43) in SCZ patients shows a limited but progressive deterioration over time which is faster than in patients with other SSDs. Results from another study suggested that mentalizing capacity may similarly decline as well (44). This may severely hamper the effects of psychotherapies such as MBT, especially concerning its impact on mentalizing, given the observed relationship between neurocognition and mentalizing (45). So, the question remains whether therapies developed for BPD can readily be transposed to SCZ.

Given the high burdens of BPD and SCZ carried by patients, their families, and society, and the potential benefits of improving mentalizing, it is crucial to better understand how treatment affects mentalizing in both disorders. Given the previously observed quantitative (37) and qualitative (39) differences in mentalizing impairment between BPD and SCZ patients, it is likely that MBT may affect mentalizing differently in both disorders.

The purpose of this study was to compare the impact of MBT on mentalizing capacity in individuals with SCZ and BPD. Mentalizing capacity was measured using a performance-based instrument before and after treatment. The changes in mentalizing capacity were compared in three groups: BPD patients who received MBT, SCZ patients who received MBT and SCZ patients who did not receive MBT.

## 2. Materials and methods

### 2.1. Study design and participants

The present study used data from two previous studies: a randomized controlled trial (30), that compared Mentalization Based Treatment for psychotic disorder (MBTp) to treatment as usual (TAU) in a sample of patients with a wide range of SSDs, and a naturalistic study with uncontrolled design that observed patients with a range of personality disorders who received MBT (34). Data of both studies were combined in order to run an explorative, comparative analysis of the effect of MBT on mentalizing capacity in patients with SCZ and BPD. Because of the substantial overlap between BPD and the broad diagnostic category of SSDs [38%; (9)], as opposed to the relatively minuscule overlap between BPD and the much more narrowly defined classification of SCZ [2%; (9)], from the original RCT sample only patients with SCZ were included (*N* = 54), not patients with other SSDs [*N* = 30; see (22)]. From the original naturalistic study (34) only participants with BPD (*N* = 28) were selected. Participants with other types of personality disorders were not included (*N* = 18). Because of the overlap between cluster A personality disorders like schizoid or schizotypal personality disorders and SCZ, no patients with a (comorbid) cluster A personality disorder were included. None of these patients had comorbid SSDs.

The current study ultimately comprised three groups of participants: 28 patients with SCZ who did not receive MBT, 26 patients with SCZ who received MBT, and 28 patients with BPD who received MBT. Patients with SCZ were recruited from community treatment teams at two mental health care facilities in the Netherlands (GGZ Rivierduinen and Altrecht) and had to meet the following inclusion and exclusion criteria: a SCZ classification [diagnosed with the Comprehensive Assessment of Symptoms and History (CASH; (46))] and not having a comorbid BPD classification; having been in treatment for SCZ from at least 6 months up to a maximum of 10 years; being between 18 and 55 years old; and not having intellectual disability or substance abuse issues (30). Participants with BPD were part of a larger group of patients with personality disorders who had been referred to the MBT team. They met the following inclusion criteria: a classification with BPD [based on Structured Clinical Interview for DSM-IV Axis II Personality Disorders (SCID-II)] and not having a co-morbid SSD, cluster A personality disorder or substance abuse issues.

### 2.2. Therapy

Both the participants with SCZ and BPD who enrolled in the MBT program, received an 18-month long treatment that consisted of psychoeducation, group therapy, individual therapy, and psychiatric consultation (and potentially psychiatric medication). MBT is a psychodynamic treatment approach drawn from attachment theory, that combines individual and group therapy. Its primary goal is to enhance mentalizing capacity, especially in stressful conditions, to decrease psychopathology and improve functioning. The MBT treatment manual [(47); was employed for both groups of disorders]. The essentials were similar, with sessions emphasizing affect in the here and now, establishing a secure therapeutic relationship, adjusting the complexity of mentalizing intervention on the basis of the level of stress, and adopting a “not-knowing” therapeutic attitude. However, disorder-specific patient characteristics necessitated different treatment approaches (30).

At the beginning of the treatment, the patients received at least four sessions that focused on teaching them about the essential components of mentalizing. The one-on-one therapy sessions provided a space where patients could discuss problems they encountered during group sessions or in their daily life, with an emphasis on five broad categories: commitment to treatment, psychiatric symptoms, social interactions, harmful or evasive behavior, and their functioning in the community. The group therapy sessions involved up to eight patients and two therapists meeting once a week for an hour.

For participants in the SCZ group, the dosage of the sessions was somewhat reduced to a 1 h group session per week and a half-hour individual session once every 2 weeks. Patients in the BPD group received individual MBT sessions once a week and group MBT sessions either once or twice a week. The decision to opt for either depended on the patient’s symptom severity and level of social functioning at baseline. The vast majority of patients (whether SCZ or BPD) received therapy from the same treatment team (the MBT unit at GGZ Rivierduinen). All clinicians involved completed a two-day MBT training program with a certified trainer in The Netherlands. To ensure treatment fidelity and adherence to the treatment manual, all therapists received weekly supervision by experienced and registered MBT supervisors who used video-taped sessions, where possible, to discuss and reflect on interventions, particularly regarding their adherence to the MBT treatment model and their contribution to mentalizing. An MBT supervisor rated four randomly selected video-taped sessions, using an MBT adherence scale, and determined them to adhere to the treatment model adequately.

### 2.3. Measures

#### 2.3.1. Mentalizing capacity

The Thematic Apperception Test (48) was used to evaluate mentalizing capacity. The TAT involves showing black-and-white pictures of ambiguous social situations to participants, who were then asked to describe what is happening in the picture and what is going through the minds of the characters. Six pictures were used. The TAT narratives were then analyzed using the Social Cognition and Object relations System [SCORS; (49)]. The SCORS assesses four dimensions of mentalizing: complexity of mental representations of people and understanding of social causality, considered to be cognitive aspects of mentalizing, as well as affect-tone of relationships, and capacity for emotional investment, which capture affective aspects of mentalizing.

Complexity of representations represents an individual’s capacity to differentiate between the perspectives of different individuals, including themselves and others, in a clear manner. It assesses whether the individual has the ability to create a psychological portrait of various individuals, depicting their motivations, emotions, behaviors, thoughts, desires, and motives, with a certain level of consistency over time. Understanding of social causality means the ability to provide a logical and psychologically minded explanation for the behavior of another. This dimension examines the accuracy and logical coherence of cause-and-effect relationships in interpersonal relations, as well as the identification of psychological mechanisms mediating between stimuli and responses. The narratives can range from being illogical, incoherent, and lacking causality to describing the psychological processes underlying behaviors and interactions. In other words, individuals react to the external world based on their intrapsychic motivational processes. This dimension measures whether the actions described in the narratives can be logically understood, meaning whether behaviors have a clear and logical cause, and whether these causes are psychologically mediated. Affect-tone measures the degree to which others are perceived as either benign or malign. The dimension measures the emotional quality of these representations within interpersonal relationships. It investigates to what extent an individual has positive or negative expectations toward others and how others are expected to respond emotionally and behaviorally. Can others be trusted, are they inclined to engage in fulfilling relationships, or provide help and comfort? In essence, are relationships enriching or do they solely elicit painful feelings? Capacity for emotional investment measures the extent to which relationships with others are perceived as inherently meaningful or merely as a means to an end. This dimension represents the capacity to invest emotionally in others and the quality of conscience. This dimension aims to assess the extent to which others are used for personal purposes or, at the opposite extreme, are respected for their autonomy and authenticity.

Each dimension is scored on a 5-point scale, with higher scores indicating better social cognitive functioning. Luyten et al. (50) emphasized the significance of the SCORS test as it incorporates almost all facets of mentalization, encompassing cognitive mentalizing, which is evaluated by complexity and comprehension of social causality, and affective mentalizing, measured through the affect tone and emotional investment dimensions. The SCORS test is a valid and dependable tool for assessing social cognition (51), with substantial consistency between pictures (52) and high inter-rater reliability (51, 52). Narratives were scored by psychology master’s students who were either blind to the experimental condition of the study (30) or unaware of whether participants had started or ended treatment (34). Interrater reliability was assessed by means of recorded narratives and rated independently by all raters. Inter-rater reliability was acceptable for complexity of representations and understanding of social causality (Cronbach’s α = 0.7), good for affect-tone of relationships (Cronbach’s α = 0.8), and excellent for capacity for emotional investment (Cronbach’s α = 0.9).

#### 2.3.2. Positive symptoms

For descriptive purposes only, positive symptoms were measured at baseline in the two SCZ groups. The Dutch translation (53) of the Positive and Negative Syndrome Scale [PANSS; (54)] was used. The score comprises the average of seven items scored on a 7-point Likert-scale. Further details can be found in Weijers et al. (55).

### 2.4. Statistical analyses

Repeated measures analyses were used to compare differences in mentalizing capacity pre-and posttreatment. Differences were analyzed for each dimension of mentalizing capacity and for each group of patients separately. ANCOVAs were used to compare differences between groups in mentalizing capacity post-treatment, corrected for baseline differences. All analyses were conducted on the basis of the intention-to-treat principle. BPD patients with MBT were compared to SCZ patients with MBT, and similar analyses comparing SCZ patient with MBT to SCZ patients without MBT were conducted. Analyses were performed with IBM SPSS Statistics for Windows (version 24).

### 2.5. Handling of missing data

The analyses of the outcomes were carried out with multiply imputed data, allowing for the use of a proper ‘intention-to-treat’ analysis. The methods for imputation were identical to the original studies [see (22, 23) for details]. To create imputed datasets, a fully conditional Markov chain Monte Carlo (MCMC) approach was used, generating five datasets for each analysis. Rubin’s rules were applied to combine the results obtained from the analyses conducted with these imputed datasets. In the group of SCZ patients who did not receive MBT 25% of data (*N* = 6) was imputed; in the group of SCZ patients who received MBT 35% of data (*N* = 9) was imputed; and in the group of BPD patients who received MBT 21% of data (*N* = 6) was imputed.

## 3. Results

### 3.1. Sample statistics

There were no differences between SCZ patients who received MBT and those who did not regarding age, gender, duration of illness, use of medication, level of education, or severity of psychotic symptoms at baseline (all *p*s > 0.09).

No differences were observed between BPD patients who received MBT and SCZ patients who received MBT on age, or level of education. There was a significant difference on gender, with a minority of patients with BPD being male (31.3%, *N* = 10), and the majority of SCZ patients being male (66.7%, *N* = 16), χ^2^(1) = 6.54, *p* = 0.01. For more demographics, please refer to Table 1.

**Table 1:** Means and standard deviations for descriptive variable in three patient groups.

	SCZ		SCZ-MBT		BPD	
	*M*	SD	*M*	SD	*M*	SD
Positive symptoms	10.77	4.09	13.33	4.52	n/a	n/a
Years since first psychosis	5.4	2.99	6.7	3.63	n/a	n/a
Dose	63.7	152.21	107.05	148.82	n/a	n/a
Age	32.4	9.35	32.8	7.92	31.07	8.84
Level of education	4.46	1.43	4.58	1.36	4.71	1.08
	***N***	**%**	***N***	**%**	***N***	**%**
Gender (male)	22	78.57	16	61.54	8	28.57

### 3.2. Time effects

Post-treatment, BPD patients with MBT scored higher on several measures of mentalizing compared to baseline, including: complexity of representations [*F* (1, 26) = 15.43, *p*_pooled_ < 0.001], understanding of social causality [*F* (1, 26) = 43.51, *p*_pooled_ < 0.001] and capacity for emotional investment [*F* (1, 26) = 10.57, *p*_pooled_ < 0.01]. No significant differences were found regarding affect-tone of relationships [*F* (1, 26) = 0.34, *p*_pooled_ = 0.63].

Post-treatment, SCZ patients with MBT did not score significantly higher on several measures of mentalizing compared to the start of treatment, including: complexity of representations [*F* (1, 26) = 1.11, *p*_pooled_ = 0.37], understanding of social causality [*F* (1, 26) = 0.76, *p*_pooled_ = 0.45] and affect-tone of relationships [*F* (1, 26) = 1.53, *p*_pooled_ = 0.34]. There was a significant decrease in capacity for emotional investment [*F* (1, 26) = −13.09, *p*_pooled_ = 0.02].

Post-treatment, SCZ patients without MBT did not score significantly higher on several measures of mentalizing compared to the start of treatment, including: complexity of representations [*F* (1, 26) = 1.65, *p*_pooled_ = 0.23] and affect-tone of relationships [*F* (1, 26) = 0.57, *p*_pooled_ = 0.64]. There was a significant decrease in capacity for emotional investment [*F* (1, 26) = −16.69, *p*_pooled_ < 0.001] and understanding of social causality [*F* (1, 26) = −12.39, *p*_pooled_ = 0.004].

### 3.3. Group vs. time interaction effects

Post-treatment, BPD patients with MBT scored higher than SCZ patients with MBT on complexity of representations [*t* (52) = 6.43, η_p_^2^ = 0.50, *p*_pooled_ < 0.001], understanding of social causality [*t* (52) = 4.94, η_p_^2^ = 0.41, *p*_pooled_ < 0.001] and capacity for emotional investment [*t* (52) = 3.26, η_p_^2^ = 0.16, *p*_pooled_ = 0.002]. No significant differences were found regarding affect-tone of relationships [*t* (52) = 0.93, η_p_^2^ = 0.04, *p*_pooled_ = 0.36].

Post-treatment, SCZ patients with MBT scored higher than SCZ patients without MBT on understanding of social causality [*t* (52) = 2.52, η_p_^2^ = 0.12, *p*_pooled_ = 0.01]. However they did not score higher on complexity of representations [*t* (52) = 1.40, η_p_^2^ = 0.06, *p*_pooled_ = 0.17], capacity for emotional investment [*t* (52) = 0.57, η_p_^2^ = 0.02, *p*_pooled_ = 0.58] or affect-tone of relationships [*t* (52) = 0.46, η_p_^2^ = 0.02, *p*_pooled_ = 0.65].

Pooled means and standard deviations at baseline and post-treatment for each subgroup of patients are shown below in Table 2.

**Table 2:** Pooled means and standard deviations for each dimension of mentalizing capacity in three patient groups.

	*N*	Baseline		18 months		Baseline		18 months	
		*M*	SD	*M*_pooled_	SD_pooled_	*M*	SD	*M*_pooled_	SD_pooled_
		Complexity				Social causality			
BPD with MBT	28	2.07	0.64	2.57	0.36	1.85	0.32	2.44	0.33
SCZ with MBT	26	2.06	0.21	2.00	0.18	1.97	0.42	1.90	0.32
SCZ no MBT	28	1.98	0.28	1.92	0.16	1.91	0.33	1.65	0.32
		**Affect-tone**				**Emotional investment**			
BPD with MBT	28	2.94	0.43	2.93	0.44	1.45	0.49	1.79	0.41
SCZ with MBT	26	2.95	0.47	3.09	0.55	1.76	0.46	1.48	0.33
SCZ no MBT	28	3.07	0.34	3.10	0.51	1.77	0.46	1.41	0.41

#### 3.3.1. Secondary analyses

Given the significant difference of gender between the BPD and SCZ groups who receive MBT, we conducted additional sensitivity analyses, to control for the potential influence of gender. The analyses were similar to the main ANCOVAs, but with gender as an added covariate. Results revealed no deviations from the results of the primary analyses. When controlling for gender, patients with BPD scored higher on complexity of representations, capacity for emotional investment and understanding of social causality (all *p*s_pooled_ < 0.006), but not on affect-tone of relationships (*p*_pooled_ = 0.25).

## 4. Discussion

This study investigated the impact of MBT on patients with BPD and SCZ in terms of mentalizing capacity. The results indicate that BPD patients who received MBT show greater improvement in mentalizing capacity in three domains compared to SCZ patients who received treatment, namely: complexity of representations, understanding of social causality, and capacity for emotional investment. In turn patients with SCZ who received MBT performed better on understanding of social causality than patients with SCZ who did not receive treatment, but not on the other domains. Results show that mentalizing capacity improved in most domains after MBT in the BPD group, which echoes earlier findings (28). However, patients with SCZ saw a decline in two of the domains of mentalizing—namely capacity of emotional investment and complexity of representations—corroborating the conclusion that mentalizing capacity may show a progressive decline in the course of the disorder (44). While the group of SCZ patients who received MBT maintained the baseline level of understanding of social causality, those who did not showed a progressive decline.

While the positive impact of MBT on mentalizing capacity in BPD is undeniable, its impact in patients with SCZ is less clear-cut. SCZ patients who received MBT showed either a stabilization (with regard to affect-tone of relationships and understanding of social causality) or a reduction (with regard to capacity for emotional investment and complexity of representations) in mentalizing capacity. However, this does not mean that MBT is ineffective in the group of SCZ patients. The results showed that the post-treatment difference on understanding of social causality between SCZ patients who received MBT and those that did not, was medium- to large-sized. Such an effect cannot easily be dismissed, even if MBT only seemed to be able to thwart the natural decline in this domain of mentalizing. Secondly, this result is believed to be meaningful as several previous studies observed a strong relationship between cognitive, other-oriented mentalizing—which we consider understanding of social causality to be—and negative symptoms and social functioning [e.g., (56)]. This may indicate that, while MBT does not *improve* mentalizing capacity, it may offer some protection against a potentially progressive decline in other oriented, cognitive mentalizing capacity and thereby potentially against the development of negative symptoms. However, more research is needed to examine the long-term effects of MBT on both SCZ and BPD. Follow-up investigations are currently being conducted to examine whether the gains in mentalizing capacity in BPD and the stabilization in SCZ last 5 years after the end of treatment.

Potential reasons for the decline in mentalizing capacity in SCZ patients over time may be manifold. Schizophrenia is widely held to be extremely damaging to interpersonal relationships and social standing. After a psychotic episode, patients may experience significant changes in their social environment, such as losing friends, romantic relationships, or employment. Social isolation may lead to decreased exposure to social cues, resulting in reduced mentalizing capacity over time. Studies have shown that social functioning tends to decline most during the first 5 years after the onset of schizophrenia (57). These losses can be difficult to recover due to factors such as hospitalizations, negative symptoms, cognitive decline, self-stigma, and medication side-effects (29). Indeed, research has shown that social isolation is associated with poorer social cognition in patients with schizophrenia (58). In a previous study we also observed that, at the end of MBT treatment, patients with a relatively recent onset SSD, functioned at a level in-between healthy controls and chronic SCZ patients (30), suggesting that patients with SCZ (or at least a more chronic SSD) are more likely to suffer from social isolation. In this regard, Fonagy and Allison (59) have suggested that the success of MBT lies in the rekindling of motivation to again engage in meaningful communication with the social environment. This, in turn, can help patients to modify their cognitive models based on feedback from others. However, patients with schizophrenia often have smaller and more fragile social networks, which may limit their ability to benefit from these interactions. As a result, they may struggle to learn from others between sessions and have poorer treatment outcomes (60, 61).

Relatedly, negative symptoms, such as affective flattening, and avolition, which may lead to decreased motivation and interest in social interactions, may result in reduced mentalizing capacity over time and can lead to decreased motivation and interest in social interactions. Indeed, research has shown that negative symptoms are associated with poorer mentalizing in patients with SCZ (56, 62).

Neurocognitive deficits, such as impairments in attention, working memory, and executive functioning, may also impact mentalizing capacity over time as research has shown that neurocognitive deficits are associated with poorer social cognition (62) and a recent study showed that the neurocognitive decline in SCZ averages about 16 IQ points over time (43).

Chronic stress is also a common feature of SCZ that can have negative effects on brain function and cognitive performance. Chronic stress can cause neuroinflammation and oxidative damage to neurons, disrupting neural networks, potentially leading to impairments in cognitive domains including mentalizing capacity (63).

### 4.1. Strengths and weaknesses

Importantly, some caveats apply to the conclusions of the current study. First, one significant weakness of the present study is that the experimental samples were derived from two previous studies with different study designs, which were not originally intended to compare SCZ and BPD. As such, the study is merely explorative in nature. Additionally, there was no BPD control group without MBT to compare to, making it impossible to accurately gage the actual impact of MBT on this group. As such, no causal conclusions can be derived from this study, and its results should be interpreted with caution.

Second, as mentioned in the introduction, a decline in neurocognitive capacity may have contributed to differences in treatment effect, however since no IQ-testing was done, it is difficult to determine how well the groups were matched at baseline on a neurocognitive level. However, we were able to determine that the three groups did not significantly differ from each other in terms of level of education and while other factors influence academic performance as well, there is a highly significant relationship between academic performance and IQ (64).

Third, the original RCT examining MBT for a wide range of SSDs (30) included more measures of mentalizing capacity including theory of mind and insight. Both were positively impacted by MBT. However, since these measures were not present in the naturalistic study of MBT in a range of personality disorders (23) we could not compare them. Still this makes it likely that there are other aspects of mentalizing that are differently affected, even in SCZ.

Fourth, the comparison between the two diagnostic groups was somewhat lop-sided. BPD patients received more MBT than the SCZ patient, with one to two group sessions per week and one individual session per week. Based on clinical experience, when initially designing the study (55), we had expected weekly individual sessions to be too strenuous for patients with SCZ. However, other authors have experienced that MBT can in fact be provided more often, even up to multiple (individual) sessions per week (37), although it remains uncertain whether this also goes for the combination of group and individual therapy. Thus, we cannot rule out that the difference in dosage of treatment may have added to the observed differences in impact. Individual sessions once per 2 weeks may have failed to instantiate a secure working relationship between client and therapist or may have resulted in too big a timespan between sessions to maintain focus on therapeutic goals. This may also have resulted in a loss of interest or motivation. More research is needed to determine whether increasing the number of sessions per week, results in more treatment success. Additionally, it is unclear what the optimal ratio of group to individual sessions is.

Fifth, this study had an attrition rate of between 21 and 35% which may have impacted the results due to selective drop-out. We tried to mitigate the impact of potential statistical artifacts caused by selective drop-out (e.g., those who are most severely affected may be most likely to drop out) with imputed data, but multiple imputation itself is held to be less reliable with greater drop-out numbers. Still, recent research has shown that even very high rates of missing data (up to 50%) can be handled adequately by multiple imputation (65).

The study’s strength first lies in its rigorous research design with blinded raters. Second, missing data were imputed enabling us to conduct a true intention-to-treat analysis. Third, the vast majority of patients received treatment by the same MBT team, at GGZ Rivierduinen, increasing internal treatment consistency between diagnostic groups. Fourth, all therapists underwent intensive supervision to ensure that sessions met MBT standards. Also, the same supervisors were involved in both diagnostic groups and across treatment facilities. This means that differences in tone and approach were kept to a minimum. Fifth, the different groups of patients were paired well on variables such as age and level of education and also on severity of symptoms and use of medication (regarding the SCZ groups). There was a significant difference in gender between the BPD group and SCZ group with MBT, but we were able to conduct a sensitivity analysis with gender as a covariate, and observed that the result did not differ significantly from the main analyses.

### 4.2. Recommendations

As our results suggest that BPD and SCZ are divergently impacted by MBT, we recommend continuing to develop a variant of mentalization-informed treatment more specifically tailored to SCZ. Previously (30), we argued that MBT for psychotic disorders should be implemented earlier rather than later during the development of the disorder as more chronic patients may benefit less from therapy than early-onset patients. The current study corroborates this view, as it suggests that the progression of SCZ, the stage of chronic psychotic vulnerability, may be characterized by a gradual decline of mentalizing capacity. Still, MBT for SCZ should not be easily dismissed, as this study also provided evidence that MBT has a medium to large stabilizing effect on other oriented, cognitive mentalizing in patients with SCZ.

Bateman et al. (66) have suggested a staged approach to the treatment of psychosis, where mentalization-informed treatment interventions should be tailored to the needs of each developmental stage of SSD. We agree and would like to add that treatment goals could also be adjusted to the developmental stage of the SSD as well. Treatment in the early stages should be aimed at increasing mentalizing capacity, prevention of onset of psychosis and the establishment of a supportive and mentalizing network around the patient. Once a first episode has occurred, the aim should be a prevention of relapse, the establishment of social support and societal rehabilitation. Lastly, concerning MBT for SCZ then, we hold that treatment perhaps should be aimed more at consolidation of (certain aspects of) mentalizing and the social network, rather than improvement, but more research is needed to substantiate this view. Additionally, as suggested elsewhere (30) we believe that MBT for SCZ should be given for a longer period of time, as it takes SCZ patients more time to feel secure enough to start exploring feeling states. For more in-depth recommendations regarding approach and technique, please refer to Weijers et al. (67).

## 5. Conclusion

The results of this study suggest that MBT improves mentalizing along multiple domains in patients with BPD. Results also suggest that mentalizing shows a limited but progressive decline in patients with SCZ without targeted treatment. MBT for patients seems to stymie the decline of mentalizing in SCZ patients, at least with regard other-oriented, cognitive mentalizing.

## Data availability statement

The original contributions presented in the study are included in the article/supplementary materials, further inquiries can be directed to the corresponding author.

## Ethics statement

The studies involving humans were approved by Medisch Ethische Toetsingscommissie Maastricht University. The studies were conducted in accordance with the local legislation and institutional requirements. The participants provided their written informed consent to participate in this study.

## Author contributions

JW was responsible for data collection and conceived the idea for the manuscript, and wrote the first and final draft. FK provided feedback and wrote the second draft. CK, RW, and J-PS provided feedback. All authors contributed to the article and approved the submitted version.

## Conflict of interest

The authors declare that the research was conducted in the absence of any commercial or financial relationships that could be construed as a potential conflict of interest.

## Publisher’s note

All claims expressed in this article are solely those of the authors and do not necessarily represent those of their affiliated organizations, or those of the publisher, the editors and the reviewers. Any product that may be evaluated in this article, or claim that may be made by its manufacturer, is not guaranteed or endorsed by the publisher.

## Footnotes

^1^Rizzi E, Weijers J, ten Kate C, Selten JP. Mentalization-based treatment for a broad range of personality disorders: A naturalistic study. *BMC Psychiatry*. Under review [preprint].
